# Data on clinical significance of GAS2 in colorectal cancer cells

**DOI:** 10.1016/j.dib.2016.05.010

**Published:** 2016-05-11

**Authors:** Chun-Chao Chang, Chi-Cheng Huang, Shung-Haur Yang, Chih-Cheng Chien, Chia-Long Lee, Chi-Jung Huang

**Affiliations:** a Division of Gastroenterology and Hepatology, Department of Internal Medicine, Taipei Medical University Hospital, Taipei, Taiwan; b Division of Gastroenterology and Hepatology, Department of Internal Medicine, School of Medicine, College of Medicine, Taipei Medical University, Taipei, Taiwan; c School of Medicine, Fu Jen Catholic University, New Taipei, Taiwan; d Breast Center, Cathay General Hospital, Taipei, Taiwan; e School of Medicine, Taipei Medical University, Taipei, Taiwan; f Department of Surgery, Taipei-Veterans General Hospital, Taipei, Taiwan; g School of Medicine, National Yang Ming University, Taipei, Taiwan; h Department of Anesthesiology, Cathay General Hospital, Taipei, Taiwan; i Department of Internal Medicine, Cathay General Hospital, Taipei, Taiwan; j Department of Medical Research, Cathay General Hospital, Taipei, Taiwan; k Department of Biochemistry, National Defense Medical Center, Taipei, Taiwan

## Abstract

The growth arrest-specific 2 (GAS2) was cloned and found to be upregulated in the feces of recurrent CRC patients. This overexpressed GAS2 induced different patterns of gene expressions in CRC cells. Briefly, one cell proliferation marker, Ki-67 antigen (Ki-67), was upregulated in the cells with overexpressed GAS2, “Correlation between proliferation markers: PCNA, Ki-67, MCM-2 and antiapoptotic protein Bcl-2 in colorectal cancer” [1]. Whereas, the expression of another cell proliferation marker, proliferating cell nuclear antigen (PCNA), changed insignificantly [1]. In addition, the mRNA level of one cyclin involving in both cell cycle G1/S and G2/M transitions was also not affected by GAS2 overexpression, “Cdc20 and Cks direct the spindle checkpoint-independent destruction of cyclin A” [2]. The experimental design and procedures in this article can be helpful for understanding the molecular significance of GAS2 in SW480 and SW620 CRC cells.

**Specifications Table**

**Table:** 

Subject area	Biology
More specific subject area	Molecular medicine
Type of data	Table, figure
How data was acquired	The commercial kit, real-time PCR, SPSS 22.0 for Window software
Data format	Filtered, analyzed
Experimental factors	CRC patients with different clinical status (recurrence and nonrecurrence) were enrolled. CRC cell lines, SW480 and SW620, were collected for further transfection.
Experimental features	Fecal total RNA was purified from these CRC patients with different clinical status and microarray analyses were performed. In addition, PCNA, Ki-67, and CCNA2 were quantified by real-time PCR from SW480 and SW620 CRC cells according to their GAS2 expressions.
Data source location	New Taipei, Taiwan, Republic of China
Data accessibility	Data are presented in this article

**Value of the data**

• Expression levels of certain genes in feces were different between CRC patients without and with recurrence.
• The data would be valuable to correlate the cell proliferation markers and the expression levels of GAS2.
• The data might support the irrelevance of GAS2 in G2 phase of cell cycle.

## 1 Data

**Fig. 1:**
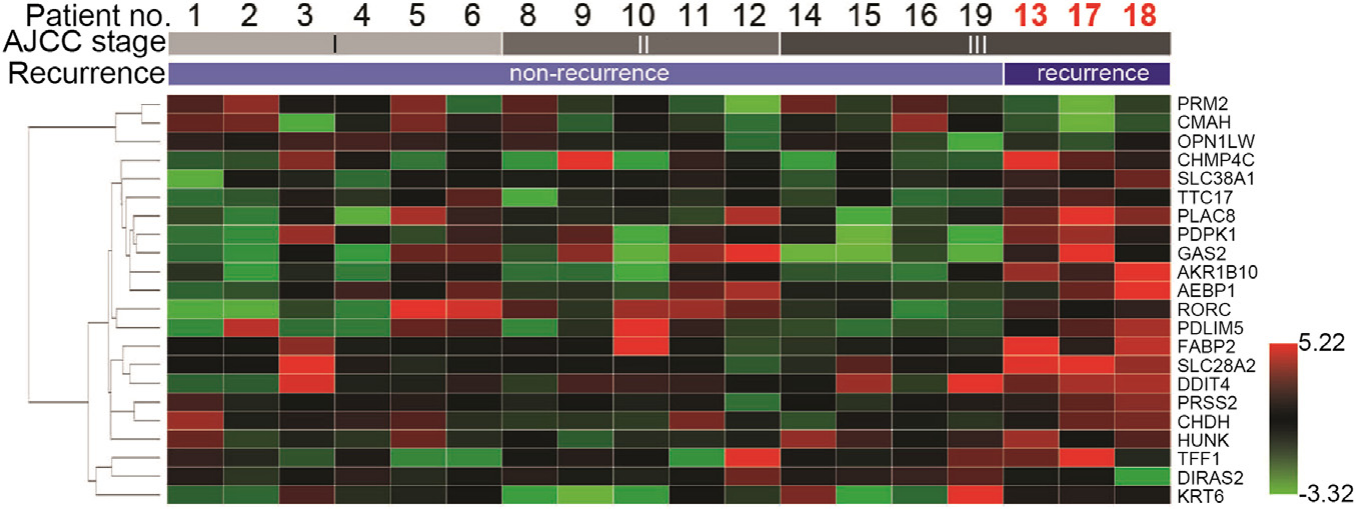
Clusters of 22 significant genes for recurrent CRC. The relative fold and statistic significance in these assays were set at >1.5-fold and *P*<0.05 on the similarity between their expressions in cases. Values of high (red) and low (green) expressions were indicated at lower right corner.

**Fig. 2:**
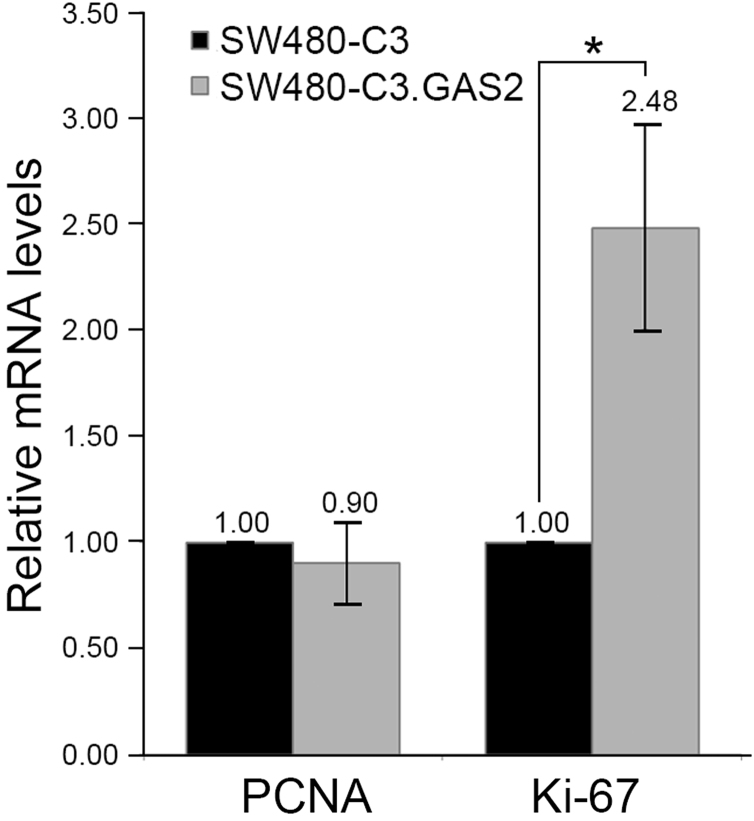
mRNA levels of proliferation markers in GAS2-overexpressed SW480 cells. Relative value of mRNA levels was indicated at bar top. SW480-C3, SW480 cells with pEGFP C3; SW480-C3.GAS2, SW480 cells with GAS2-contained pEGFP C3. PCNA, proliferating cell nuclear antigen; Ki-67, Ki-67 antigen; GAPDH, glyceraldehyde-3-phosphate dehydrogenase; GAS2, growth arrest-specific-2. SW480, ATCC CCL-228. *P<0.05.

**Fig. 3:**
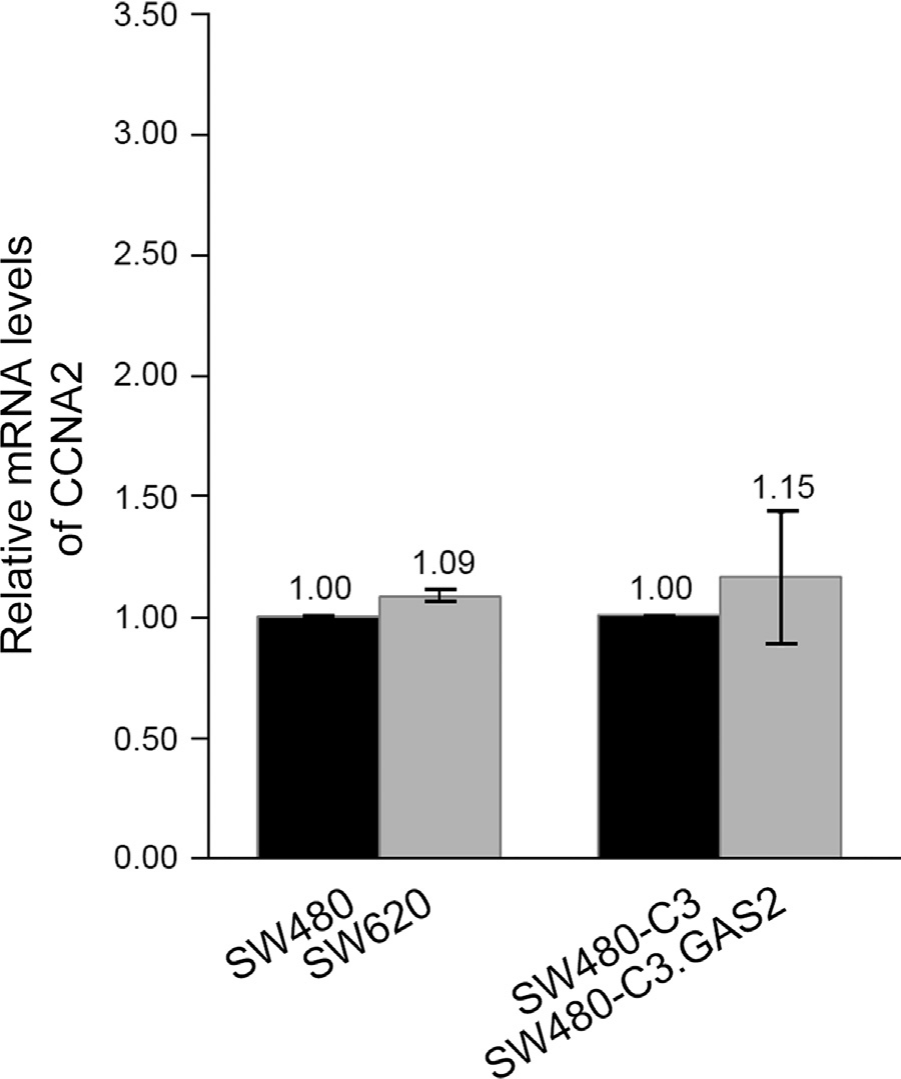
mRNA levels of CCNA2 in relation to GAS2 overexpression. Relative value of mRNA levels was indicated at bar top. SW480-C3, SW480 cells with pEGFP C3; SW480-C3.GAS2, SW480 cells with GAS2-contained pEGFP C3. CCNA2, cyclin A2; GAPDH, glyceraldehyde-3-phosphate dehydrogenase; GAS2, growth arrest-specific-2. SW480, ATCC CCL-228; SW620, ATCC CCL-227.

The data shared in this article are the experimental analyses of GAS2 expression in clinical samples, patient׳s feces, and CRC cell lines. The level of GAS2 would be up-regulated in the cases with recurrent CRC by microarray analysis (Fig. 1) [3]. The relative mRNA levels of interested genes (proliferating cell nuclear antigen: PCNA; Ki-67 antigen: Ki-67; cyclin A2: CCNA2) in different cells are presented in Figs. 2 and 3.

## 2 Experimental design, materials and methods

### 2.1 Expression difference in feces of nonrecurrent and recurrent CRC patients

Candidates in feces of recurrent patients were clustered from each comparison in relative to those fecal expressions of non-recurrent patients. The case numbers and stages in these bioinformatic analyses were 15 non-recurrent patients (6 stage I, 5 stage II, and 4 stage III) and 3 recurrent patients (stage III). Candidates in CRC tissues were extracted from GEO: GSE17536, GEO: GSE17537, GEO: GSE17538_GPL570 (Moffitt Cancer Center and Vanderbilt University Medical Center, respectively), GEO: GSE12032 (individual comparison at each stage for non-recurrent vs. recurrent), and GEO: GSE27854 at the public website of Gene Expression Omnibus (www.ncbi.nlm.nih.gov/geo) [4], [5], [6], [7]. Genes with potentially significant changes were selected by comparing the data from non-recurrent and recurrent CRC tissues. 22 genes were selected due to the cross-compared results from fecal and CRC tissue microarrays.

### 2.2 The significance of GAS2 in cell proliferation

We established two SW480 cell lines to overexpress GAS2. Firstly, GAS2 cDNA was amplified from a human placenta library (Sigma-Aldrich, St. Louis, MO, USA) (Table 1) and the polymerase chain reaction (PCR) bands for GAS2 were sequenced to confirm gene identity. Then, SW480 cells expressing GAS2 were generated either by lentiviral transduction using the all-in-one tetracycline-inducible plasmid (pAS4.1w.Ppuro-aOn) (Academia Sinica, Taipei, Taiwan) or by stable transfection using a plasmid encoding green fluorescent protein (pEGFP C3) (Takara Bio, Shiga, Japan).

The relative mRNA levels of PCNA (NM_002592) and Ki-67 (NM_002417) in cells were quantified by quantitative real-time PCR and relative to the mRNA level of GAPDH (NM_002046). As shown in Fig. 2, the level of Ki-67 in SW480-C3.GAS2 was significantly higher than that in control cells without GAS2 overexpression (SW480-C3). Primer sequences used in these gene quantifications were described as following: PCNA (universal probe, #69), 5′-TGGAGAACTTGGAAATGGAAA-3′ for forward primer and 5′-GAACTGGTTCATTCATCTCTATGG-3′ for reverse primer; Ki-67 (universal probe, #73), 5′-CAAGAGGTGTGCAGAAAATCC-3′ for forward primer and 5’-TCACTGTCCCTATGACTTCTGG-3′ for reverse primer. The expression level of GAPDH (universal probe, #60; forward primer, 5′-CTCTGCTCCTCCTGTTCGAC-3′; reverse primer, 5′-ACGACCAAATCCGTTGACTC-3′) was used as an internal control for calibration.

### 2.3 The correlation of GAS2 in the expression of CCNA2

The molecular marker for G2 phase of cell cycle, CCNA2 (NM_001237), was specific quantified by quantitative real-time PCR. The relative mRNA levels of CCNA2 were also calibrated with that of GAPDH as described before. However, no significant difference was found in the cells with overexpressed GAS2 (Fig. 3). Sequences of primer pair for CCNA2 were 5′-CCATACCTCAAGTATTTGCCATC-3′ (forward primer) and 5′-TCCAGTCTTTCGTATTAATGATTCAG-3′ (reverse primer). The number of universal was #67.

## Figures and Tables

**Table 1:** Primers for cloning growth arrest-specific 2 into pAS4.1w.Ppuro-aOn plasmid.

Official symbol	Accession	Sequence (from 5׳ to 3׳)^a^	Product size
GAS2	NM_005256	*F*: GCGATCGCGCTAGC**ATG**TGCACTGCTCTGAGCCC	964bp
		*R*: GTTTAAAC**TCA**CTTAATTTCCTTCTTAGCCT	

*Abbreviations*: GAS2, growth arrest-specific 2; *F*, forward primer; *R*, reverse primer; pAS4.1w.Ppuro-aOn, all-in-one tetracycline-inducible plasmid.

a Underline indicated the AsiSI restriction site at forward primer and the PmeI restriction site at reverse primer; start codon (ATG) and stop codon (TGA; reverse sequence: TCA) were in bold letters.
