# Supplementary material for: Data on clinical significance of GAS2 in colorectal cancer cells
Source: Data Brief. 2016 May 11;8:82–6. doi: 10.1016/j.dib.2016.05.010 (PMC4887555; doi:10.1016/j.dib.2016.05.010)
Supplement: Supplementary file 1 — Supplementary material [file mmc1.docx]

Conflicts of interest: All authors declare that they have no conflict of interest.
